# Improvement in Cognition Following Double-Blind Randomized Micronutrient Interventions in the General Population

**DOI:** 10.3389/fnbeh.2019.00115

**Published:** 2019-05-28

**Authors:** Rebecca J. Denniss, Lynne A. Barker, Catherine J. Day

**Affiliations:** Centre for Behavioural Science and Applied Psychology, Department of Psychology, Sociology and Politics, Sheffield Hallam University, Sheffield, United Kingdom

**Keywords:** micronutrient supplements, cognition, healthy adults, 8-week intervention, diet

## Abstract

The impact of poor nutrition on physiological health is well understood (Costarelli et al., 2013). Less is known about the effects of diet on brain function and cognition in the general population (Ames, 2010; Parletta et al., 2013; White et al., 2017) and we are still in the early stages of understanding the role of specific nutrients to normal and pathological neuronal functioning. In the present study, the putative effect of a multivitamin/mineral or vitamin D supplement on cognitive function over an 8-week period was compared with volunteers taking vitamin C. Healthy adults (*N* = 60) were recruited, age range 21–59 years ($\bar{x}$ = 39.07 years, SD = 11.46), with participants randomly allocated to conditions in a double-blind protocol. Participants also completed a 14-day food diary to gather information on micronutrient intake. The cognitive test battery included measures from the Wechsler Adult Intelligence Scale-III (WAIS-III; Wechsler et al., 2008), Wechsler Memory Scale-IV (WMS-IV; Wechsler, 2009) and Delis-Kaplan Executive Function System (D-KEFS; Delis et al., 2001), along with the Doors and People (Baddeley et al., 1994) and a serial reaction time task. Analyses showed better performance on some tasks in all groups following the intervention period, notably on measures of verbal and visual memory and visuomotor processing speed. The Multivitamin group showed significant improvements on tasks of visual strategy generation (along with the Vitamin C group), motor planning, explicit and implicit learning, and working memory. This evidence suggests that sub-optimal micronutrient intake may have a negative effect on cognition across the lifespan.

## Introduction

Micronutrients (vitamins and minerals) are essential for physical and cognitive health (Kennedy and Haskell, 2011; Turner, 2011; Bailey et al., 2015). The impact of poor nutrition on physiological health is well understood (Costarelli et al., 2013), but less is known about the effects of diet on brain function and cognition in the general population (Ames, 2010; Parletta et al., 2013; White et al., 2017). Many nutrients play an important role in brain functioning including cellular energy production, myelin generation, cell maintenance and repair, and neurotransmitter synthesis (Bourre, 2006; Harms et al., 2011). They must be acquired through diet or supplements as the human body is unable to synthesize sufficient vitamins and minerals to meet requirements (Pittas et al., 2010; Ward, 2014).

Increased consumption of convenience and nutritionally poor food may result in micronutrient deficiency, termed “hidden hunger” (Travis et al., 2004; Ruxton, 2011). Macronutrients (protein, carbohydrates, fats) in the diet are the main source of calorie intake, however, micronutrients underpin cellular metabolic processes and normal development (Ames, 2006; Bourre, 2006). Individuals with poor diet may meet or exceed kilocalorie intake required to meet energy needs, but food eaten may not be rich enough in micronutrient content for optimal cellular function. In addition, limited sunlight in the northern hemisphere makes vitamin D deficiency common in the normal population, with research indicating that 25% of the general population have serum vitamin D levels in the insufficient range in summer months, increasing to 60% of the population in the winter (Webb et al., 2010). Low vitamin D status has been associated with depression (Anglin et al., 2013), poorer cognitive function, cognitive decline and greater risk for Alzheimer’s disease compared to those with adequate or high vitamin D plasma levels (Keeney and Butterfield, 2015; Miller et al., 2015), emphasizing the important role of this nutrient for normal cognitive function. Spencer et al. (2017) recently proposed that there is scope for dietary intervention, particularly with micronutrients, to ameliorate the adverse effects of poor diet on cognition. They concluded that understanding the interplay between diet, emotion and cognition is crucial to revealing mechanisms involved in cognitive decline in aging.

We are still in the early stages of understanding the role of specific nutrients to normal and pathological neuronal functioning, although evidence suggests myriad adverse effects on brain cell structure and function, including poor mitochondrial efficacy and DNA stability associated with poor micronutrient intake (Ferguson et al., 2015; Sverdlov et al., 2015). Mitochondria are the cellular battery driving all of the cell’s functions and DNA codes for cellular proteins that metabolize nutrients and perform cellular functions.

Research into degenerative diseases of aging has linked mitochondrial aging and DNA damage caused by micronutrient deficiency to increased incidence of cognitive decline and stroke (among other diseases) in the general population (Spencer et al., 2017). These include Alzheimer’s disease, Parkinson’s disease, multiple sclerosis, autism spectrum disorders, depression, fatigue and schizophrenia (Oudshoorn et al., 2008; Balion et al., 2012; Bitarafan et al., 2014). This is of particular concern in individuals consuming food rich in fats and carbohydrates but poor in micronutrient content (Ames, 2006, 2010).

Research has shown that B-complex vitamins (including thiamine, niacin and pyridoxine) are important for cell metabolism, neurotransmitter biosynthesis, and myelin sheath integrity (Molina et al., 2012; Parletta et al., 2013). Both vitamins C and E are antioxidants that counteract the toxic effects of reactive oxygen species and lipid peroxidation produced during normal cellular function (Traber and Stevens, 2011). Insufficient quantities of these micronutrients result in damage to previously healthy cells (Montezano and Touyz, 2012). Calcium is vital for mitochondrial maintenance, gene expression and cellular calcium homeostasis (Catterall, 2011). Iron contributes to oligodendrocyte development, the glial cells responsible for axonal myelination (Todorich et al., 2009), and zinc modulates neurotransmission and contributes to the health of synapses (Gower-Winter and Levenson, 2012). Consequently, sub-clinical deficiency in micronutrient availability may have deleterious consequences for neurons and neuronal functions—perception, cognition and memory for example.

Findings from multivitamin and single nutrient studies have been mixed and there is little consensus on the benefits of micronutrient supplementation on cognitive function (Buell et al., 2009; Grima et al., 2012; Kennedy, 2016). Reviewed data showed that omega-3 fatty acids, B group vitamins and vitamin E supplements had no demonstrable effect on cognition in healthy older adults over 40 with normal or mildly impaired cognition (Forbes et al., 2015). However, normal dietary intake was not evaluated alongside the intervention. A meta-analysis of 10 randomized placebo-controlled trials in healthy cohorts aged 18–86 years showed no significant improvement in attention, visual processing or simple reaction time ability over 3–6 months supplementation periods (Grima et al., 2012), although findings showed improved memory associated with levels of micronutrients in supplements (Grima et al., 2012).

In contrast, other studies found an association between multivitamin supplementation, faster and more accurate numerical processing in healthy adults (Haskell et al., 2010; Kennedy et al., 2010), improved choice reaction time in children (Haskell et al., 2008), and improved immediate verbal memory and fluency in cognitively healthy older adults (Grima et al., 2012) compared to baseline performance. The picture is far from clear however as contrasting findings have been shown with the same supplement (Swisse Men’s Ultivite) in similar cohorts (males between 50 and 74 years), using the same computerized cognitive battery (Pipingas et al., 2010, 2014; Harris et al., 2012). Pipingas et al. (2014) found improvement from baseline in selective attention and response inhibition that trended toward significance following 16 weeks of supplementation, whereas Harris et al. (2012) found significant improvements from baseline in recognition memory after 8 weeks of supplementation. Despite the differences in length of supplement period both studies found significant change in blood plasma levels of B_6_, B_12_, folate and homocysteine (Harris et al., 2012; Pipingas et al., 2014) indicating that changes in blood levels and cognitive function can be seen over a relatively short 8 week period of supplementation. These findings are encouraging because they indicate that even brief nutritional enhancement can significantly elevate circulating micronutrient levels, presumably with beneficial effects for health, although it is less clear why different elements of cognition were altered in the different studies.

In summary, it is unclear from the literature whether brief micronutrient supplementation has benefits on brain health and subsequent cognitive function in an otherwise normal sample. In the present study, we investigated the putative effect of a multivitamin/mineral supplement or vitamin D supplement alone on cognitive function over an 8-week period in healthy volunteers compared with volunteers taking vitamin C, with all participants tested at baseline and post-intervention.

The design enabled comparative analyses of results from both single vitamin and multivitamin intervention using baseline and follow-up cognitive tests. We measured memory, higher cognitive (executive) functions and mood state, functions previously demonstrated to be negatively affected by micronutrient insufficiency (e.g., Wilkins et al., 2006; Buell et al., 2009; Kesse-Guyot et al., 2011; Chiplonkar and Kawade, 2014).

## Materials and Methods

This study was approved by Faculty Research Ethics at Sheffield Hallam University FREC 192014 and is registered with ClinicalTrials.gov (NCT03032302) as the first in a series of studies. This normative study forms part of a larger research programme investigating putative effects of nutrition on normative and neuropathological (traumatic brain injury) groups.

### Participants

Healthy participants (*N* = 61) were recruited to the study, the age ranged from 21 to 59 years ($\bar{x}$ = 39.07 years, SD = 11.46; 75% female). Sixty participants completed the study; one was lost to attrition on physician’s orders following medical diagnosis. An equal number of participants were assigned to each group; vitamin D (*n* = 20, 85% female), multivitamin (*n* = 20, 60% female) and vitamin C (*n* = 20, 80% female). The ideal sample size for a normative cohort is considered to be around 70 participants in line with recommendations (Teare et al., 2014). Sixty participants completed the study with an even number in each group so we slightly under recruited. However, our cohort size compares favorably to other recent studies, for example please see recent studies (Harris et al., 2011; *N* = 50, Macpherson et al., 2012; *N* = 41, Scholey et al., 2013; *N* = 25, von Arnim et al., 2013; *N* = 42, Whyte et al., 2016; *N* = 24).

Inclusion Criteria: able to give informed consent, aged between 18 and 60 at the time of recruitment, normal dietary requirements and intake, corrected to normal vision, no reported perceptual deficits, no history of significant head injury or diagnosis of neurodegenerative disease.

Exclusion Criteria: aged below 18 or over 60, unable to give informed consent, required a special diet, diabetic, history of head injury, pregnant or breast feeding. Individuals taking vitamin or mineral supplements at recruitment or who had taken supplements in the previous 4 weeks were also excluded.

We specifically aimed to have *minimal* exclusion criteria because we wanted to recruit a population that was as representative as possible of a “normal” cohort and extensive exclusion criteria can introduce recruitment bias.

There were no statistically significant differences in age across the three groups (*F*_(2,57)_ = 0.69, *p* = 0.505, *η*^2^ = 0.024). Mean, standard deviation and range of ages in each group are as follows:

- Vitamin D: *M* = 37.65, *SD* = 12.42, range = 21–59 years,
- Multivitamin: *M* = 41.50, *SD* = 9.43, range = 23–57 years,
- Vitamin C: *M* = 37.95, *SD* = 12.46, range = 22–58 years.

All participants were living in South Yorkshire and North East Derbyshire at the time of recruitment.

### Statistics

Food diary data were entered into a nutritional analysis software application (Netwisp version 3.0; Tinuviel Software, Llanfechell, Anglesey, UK). A mean intake value of each micronutrient was computed over the consecutive 14-day period for each participant. Analysis of variance (ANOVA) compared between group intake of each micronutrient. A difference score was calculated comparing mean intake of each micronutrient and recommended reference dietary intake (RDI) levels, as issued by the United States Food and Nutrition Board of the Institute of Medicine (Institute of Medicine (US) Standing Committee on the Scientific Evaluation of Dietary Reference Intakes, 1997, 1998; Monsen, 2000; Trumbo et al., 2001; Del Valle et al., 2011). Analyses of intake was conducted on individual micronutrients as recommended daily amounts vary. This difference score was then used to calculate a percentage above or below RDI of each micronutrient from food diary data alone and for food diary data plus intake with additional supplements for each group.

We measured IQ (WASI-II), visual and verbal memory (Logical Memory and Visual Reproduction from WMS-IV; Doors and People), visual working memory (Symbol Span from WMS-IV), executive (higher) function (Symbol Search from WAIS-IV; Verbal Fluency, Design Fluency, Trail Making, and Tower from D-KEFS) and mood state (Positive and Negative Affect Schedule) at baseline and post-intervention. Cognitive test measures were scored and age-scale adjusted with reference to test manuals. Outliers were removed or adjusted in line with Tabachnick and Fidell (2014). Age-normed standardized baseline cognitive data were analyzed with multivariate ANOVA (MANOVA). Data for each of the three groups were analyzed separately at post-intervention because they were nutritionally different at this point (different composition of supplements taken), so pair-wise *post hoc* comparisons were used to investigate putative changes in cognitive performance over the period of the study.

In our study, all analyses were based on intention to treat assumptions with no exclusion of participants assigned to each of the three groups (see McCoy, 2017). Intention-to-treat analysis is a recommended approach analyzing results in a prospective randomized study. All participants randomized to a group are included in the statistical analysis according to the group they were originally assigned to, regardless of intervention. This method allows the investigator to reduce risk of bias.

This study was conducted in the laboratory at the Psychology Department, Sheffield Hallam University, according to the guidelines laid down in the Declaration of Helsinki. All procedures involving human subjects were approved by the Sheffield Hallam University Faculty of Development and Society Research Ethics Committee. All participants provided written informed consent.

### Design

The current study was double-blind with a mixed 3*(2) design. We adhered to the standard and recommended approach of using a random number generator to assign participants to each study arm (random.org). Use of a random number generator is an approved method for ameliorating recruitment and allocation bias used to assign participants to groups in experimental intervention studies (see Schulz and Grimes, 2002). Participants completing a baseline set of cognitive measures before an 8-week micronutrient supplementation period, followed by re-test on the cognitive battery, participants completing alternative forms of measures where available. Raw scores were age-adjusted (by converting raw scores to age-related score) following scoring manual guidelines. Participants also completed a 14-day food diary during the intervention period. It is acknowledged within the nutrition literature that self-report food diaries are susceptible to random error and reactivity bias, however this method of recording dietary intake is less susceptible to systematic error than food frequency questionnaires and remains the Gold Standard for nutrient-based research (Ralph et al., 2011; Mak et al., 2012). In the current study, participants were instructed to complete diaries at time of eating to reduce distortion and poor accuracy associated with distance recall in line with extant recommendations (Kirkpatrick et al., 2014). Importantly, most nutrition research employs a 3-7 day food diary methodology to evaluate nutrient intake (Patel et al., 2006; Crispim et al., 2011), a much shorter timeframe than we used here. In this respect, our study is more rigorous than most and we chose to capture nutrient intake for 2 weeks as this is recommended as the optimum timeframe for optimal length of food record for capturing normal variety in eating patterns (Falciglia et al., 2009). In addition, participants in our study were instructed not to change their diet in any way but to proceed with their normal diet. Finally, one of the main purposes of the study was to assess “normal” or “usual” intake in the general population. Influencing intake *via* standardization would have introduced bias and likely prevented measurement of typical nutritional intake in this normative sample.

In relation to vitamin D and sun exposure, Vitamin D_3_ has been proven to be the more potent form of vitamin D in all primate species, including humans (Houghton and Vieth, 2006), for this reason the supplement used in this study contained vitamin D_3_. In this way, we took dermal vitamin D synthesis into consideration and selected the best nutrient on this basis.

This length of food diary provided an overview of individual micronutrient intake without invasive testing. Results were analyzed with MANOVA and pair-wise comparisons where relevant.

### Materials

#### Groups

Participants were randomly allocated to one of three conditions: 200 mg vitamin C, 10 μg vitamin D or Boots A-Z multivitamin/mineral supplement (see Supplementary Material Appendix 1 for contents and dosage).

Studies indicating a relationship between vitamin C intake and cognitive measures have been at supplementation doses of 500 mg/day or above (e.g., Arlt et al., 2012). A vitamin C supplement of less than half this amount (200 mg) was therefore selected to function as a quasi-control in this study. We did not expect this level of Vitamin C supplementation to have any effect on cognition and results are surprising.

Vitamin D_3_ at a dose of 10 μg/day (the RDA) was selected as this vitamin has been demonstrated to be more bioactive with increased affinity for the vitamin D receptor when compared to vitamin D_2_ (Houghton and Vieth, 2006). We compared multiple brands of vitamins to select one that had a complete nutritional profile for the multivitamin group. The composition of the multi-micronutrient supplement administered included 100% of the RDI of all micronutrients except for vitamin A and copper (50%), calcium and manganese (23%) and magnesium (16%).

Participants were asked to take one tablet per day with food as per manufacturer’s guidelines for the 8-week intervention. Follow-up testing occurred post-intervention. Compliance was assessed by number of tablets remaining on follow-up; those with less than 80% compliance were excluded (those with >11 tablets remaining).

#### Food Diary

Participants completed a food diary for the first 14 consecutive days during the study. Participants were instructed to continue with normal eating habits, to be specific about the amount of each item eaten and provide brand names and constituent items of dishes prepared.

Our 14-day food capture window was twice the length of most studies—that use a much smaller timeframe to capture general eating habits (typically 3–7 days, Hughes et al., 2012; White et al., 2016; Zweers et al., 2018), although our methods are similar in other respects. There was no need to repeat this, unlike physiological measures because participants were instructed to follow their normal dietary pattern (indeed this instruction was likely superfluous because it is incredibly difficult to get people to change their eating habits). Normal average dietary intake values were computed for each participant from food diary data and combined with RDA levels of relevant supplements following the standard process for nutrition supplementation food diary studies.

#### Test Battery

Several higher order (executive) and memory functions have shown improvement after dietary supplementation (Haskell et al., 2008; Buell et al., 2009; Kennedy et al., 2010; Blanton et al., 2013; Scott and Murray-Kolb, 2015; Hughes et al., 2017). We selected standardized measures designed to measure these functions. An affect measure was also included following evidence for micronutrient supplementation improving mood state in clinical and normative populations (Kaplan et al., 2007; Kennedy et al., 2010). Participants were matched for age and IQ across all conditions using the Wechsler Abbreviated Scale of Intelligence (WASI II; Wechsler, 2011).

Six test presentation orders were used to counterbalance measures and account for the potential confounds of order, practice and fatigue effects. Where alternative tests were available these were used at second test stage. Research indicates that an 8-week period between test-retest is sufficient for minimal carry-over effects (e.g., Harvey et al., 2005).

The visual and verbal subtests of the Wechsler Memory Scale (WMS-IV; Wechsler, 2009) and the Doors and People task (Baddeley et al., 1994) assessed visual and verbal memory. WMS-IV subtests required participants to remember two verbally presented stories (Logical Memory) and five visually presented single or paired diagrams (Visual Reproduction) in immediate and delayed conditions. Scores were allotted for the number of correctly recalled items in each condition. Participants also completed the Symbol Span sub-test of the WMS-IV (Wechsler, 2009) measuring visual working memory span. In this task, participants recall the position of an incrementally increasing string of visually presented abstract symbols. Scores are awarded for correct recall of the complete string in the correct order.

The Doors and People task comprises four subtests requiring participants to learn and correctly recall visually or verbally presented stimuli in immediate and delayed conditions. Scores are allotted for each correctly recalled name, door or symbol.

We assessed executive (higher) cognitive functions with the Symbol Search subtest from the Wechsler Adult Intelligence Scale (WAIS-IV; Wechsler et al., 2008). In this task, participants are required to correctly indicate if either one from a pair of abstract symbols is repeated in an associated string of symbols also present. We also measured Verbal Fluency, Design Fluency, Trail Making Tasks and the Tower Test completion from the Delis-Kaplan Executive Function System (D-KEFS; Delis et al., 2001). These executive function tasks measured strategy initiation, response inhibition, information processing speed, prospective planning, temporal sequencing and planning errors, respectively.

The Verbal Fluency task comprises three subtests; phonemic fluency, semantic fluency and semantic switching. These tasks require participants to verbalize novel responses to letters or categories by following a set of rules in a 1-min time period. Correct responses, repetitions and set-loss errors are recorded. Similarly, in the Design Fluency task participants must create novel designs using straight lines to link a series of dots following given rules in a 1-min time period.

The Trail Making task comprises five conditions: joining dots, numbers, letters and switching between the two or canceling numbers. Scores reflect the time taken to complete each task including correction of errors. The Tower task requires participants to move discs across several poles (towers) to match test configuration. Participants are required to complete the task with an increasing number of discs following a number of rules (for example, not placing a large disc on top of a small disc, not moving more than one disc at once). First move time, total number of moves, time taken to complete each configuration and rule infractions are recorded.

We measured implicit and explicit learning with a Serial Reaction Time Test used in our previous studies (Barker et al., 2006, 2010; Barker, 2012). This task was included to provide a measure of implicit and explicit learning ability sensitive to frontal lobe disruption. Participants were asked to respond as quickly as possible to the computer presentation of circles in four pre-set positions, either in a set pattern or a pseudo-random order. Implicit cognition was measured by the difference in response to the pattern vs. the pseudo-random presentation. Explicit cognition was scored in relation to the extent to which participants were able to recall the sequence that the circles followed (for a full description of this task, see Barker, 2012).

Finally, we measured mood state with the Positive and Negative Affect Schedule (PANAS; Watson et al., 1988). Participants were asked to rate the extent to which they had experienced had felt 20 emotions over the previous week on a Likert scale. The rating scale ranged from 1 (very slightly/not at all) to 5 (extremely), with higher scores reflecting greater positive or negative effect.

## Results

Participants were randomly assigned in a double-blind protocol. Results of descriptive analyses showed that groups were not significantly different either on IQ: (*F*_(2,57)_ = 0.60, *p* = 0.553, *η*^2^ = 0.021), or age (*F*_(2,57)_ = 0.69, *p* = 0.505, *η*^2^ = 0.024).

All cognitive measures were scored with reference to standardized reference data in scoring manuals (for descriptive statistics, see Table 1).

**Table 1:** Descriptive statistics of baseline cognitive task performance by group.

Cognitive measure	Group		
	Vitamin D (*n* = 20)	Multivitamin (*n* = 20)	Vitamin C (*n* = 20)
	Mean (SD)	Mean (SD)	Mean (SD)
WASI-II Full Scale IQ	113.15 (10.41)	110.40 (12.07)	114.15 (11.16)
WAIS-III Digit Span Correct Recall	10.85 (3.03)	10.15 (2.96)	11.50 (2.21)
WMS-IV Logical Memory Immediate Recall	11.60 (2.28)	10.80 (2.59)	11.75 (2.53)
WMS-IV Logical Memory Delayed Recall	11.35 (3.13)	11.00 (2.64)	11.45 (2.61)
WMS-IV Visual Reproduction Immediate Recall	12.40 (2.74)	12.25 (2.34)	12.30 (2.87)
WMS-IV Visual Reproduction Delayed Recall	11.55 (3.65)	10.95 (2.35)	13.05 (3.25)
WMS-IV Symbol Span Correct Recall	11.75 (3.04)	11.80 (3.58)	12.25 (2.95)
Doors and People Overall Score	12.35 (3.27)	12.05 (2.63)	12.65 (2.35)
DKEFS Trail Making Number/Letter Switching	12.05 (1.39)	11.75 (1.92)	12.40 (1.31)
DKEFS Verbal Fluency Phonemic Fluency	13.10 (2.55)	11.90 (3.67)	12.65 (3.47)
DKEFS Verbal Fluency Semantic Fluency	14.40 (3.07)	12.80 (3.86)	15.25 (2.69)
DKEFS Verbal Fluency Semantic Switching	14.85 (3.18)	12.85 (3.22)	14.75 (2.99)
DKEFS Design Fluency Total Correct Designs	8.25 (2.51)	7.90 (1.55)	8.35 (1.39)
DKEFS Tower Total Score	12.50 (2.74)	12.30 (2.56)	12.75 (2.17)
DKEFS Tower Mean 1st Move Time	11.50 (1.36)	10.95 (2.37)	11.70 (2.11)
WAIS-III Symbol Search Correct	12.75 (3.52)	12.05 (2.67)	13.60 (2.04)
Serial Reaction Time Test Explicit Learning	11.05 (4.47)	11.15 (5.33)	9.80 (3.32)
Serial Reaction Time Test Implicit Learning	79.52 (38.90)	64.74 (63.25)	89.22 (41.08)
Serial Reaction Time Test Implicit Learning Outliers Removed	79.52 (38.90)	81.14 (31.87)	89.22 (41.08)
PANAS Positive Affect Rating	33.60 (7.35)	36.30 (6.14)	35.50 (5.49)
PANAS Negative Affect Rating	19.50 (6.42)	17.45 (5.42)	17.65 (6.38)

MANOVA using Pillai’s Trace conducted in SPSS (IBM SPSS Statistics, version 23) found no significant difference between groups on cognitive performance at baseline, *F*_(40, 78)_ = 0.70, *p* = 0.891, *η*^2^ = 0.26.

**Figure 1:**
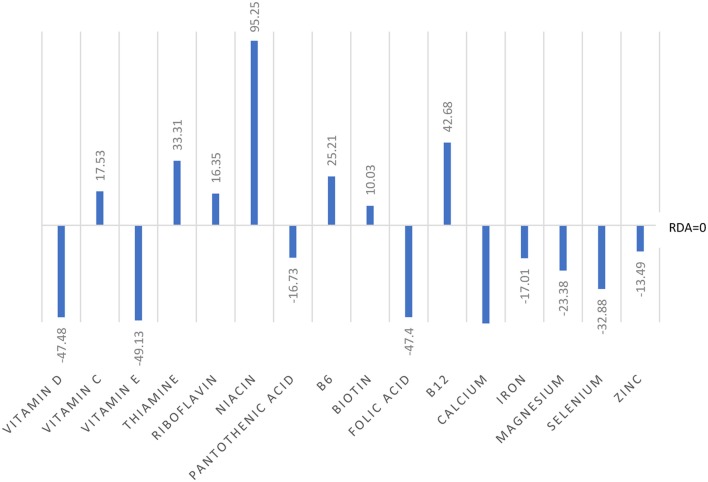
Dietary intake of micronutrients as a percentage below and above reference dietary intake (RDI) for the whole cohort (*N* = 60).

**Figure 2:**
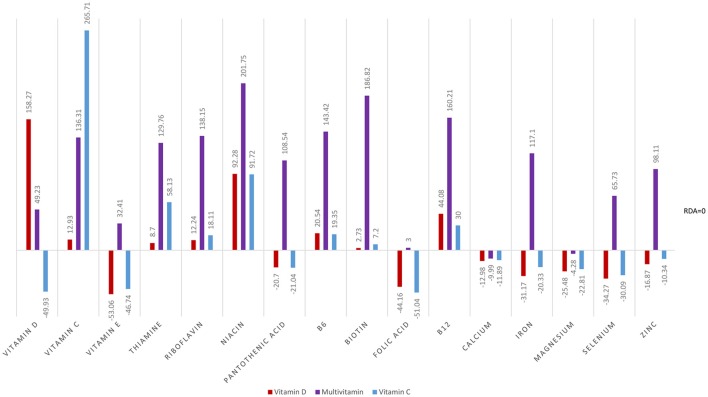
Total intake of micronutrients (dietary intake plus supplements) as a percentage below and above RDI post the supplementation interval.

Analyses of food diary data showed that for 9 out of 16 nutrients measured the total cohort (*N* = 60) had dietary intake below RDI amounts. Figural representation of dietary intake compared to RDI can be seen in Figure 1. Where RDI levels for males and females differed the RDI level for females was used as the reference point as 75% of the cohort was female. Supplementation resulted in individuals having mean intake above RDI amounts apart from for calcium and magnesium (see Figure 2).

At baseline, MANOVA using Pillai’s Trace of average daily intake (taken from food diaries) as the independent variable and group as the dependent variable showed no significant difference in dietary micronutrient intake between groups, *F*_(32,86)_ = 0.83, *p* = 0.726, *η*^2^ = 0.24. Repeat MANOVA following supplementation showed a significant effect of group on overall micronutrient intake (diet plus supplements), *F*_(32,86)_ = 76.14, *p* < 0.001, *η*^2^ = 0.97; individual between subject ANOVAs were all significant at the <0.001 level. *Post hoc* *t*-tests with correction showed the Multivitamin group had micronutrient levels significantly above both the Vitamin D and Vitamin C groups, except for vitamin D and vitamin C levels as expected. The Multivitamin group had significantly greater vitamin C intake than the Vitamin D group, *t*_(58)_ = 6.16, *p* < 0.001, and in the Vitamin C group had significantly higher intake than both the Multivitamin and Vitamin D groups (*t*_(58)_ = 6.88, *p* < 0.001; *t*_(58)_ = 17.03, *p* < 0.001, respectively).

At baseline, the whole group could not be distinguished on the basis of nutrient intake and were deficient on 9 of 16 nutrients based on RDI, the addition of a multivitamin completely changes the nutritional profile of the multivitamin group. However, with the exception of vitamins C and D, respectively, Vitamin C and Vitamin D, groups showed the same nutritional profile as baseline. Consequently, we analyzed each group’s baseline and post-intervention data separately.

**Figure 3:**
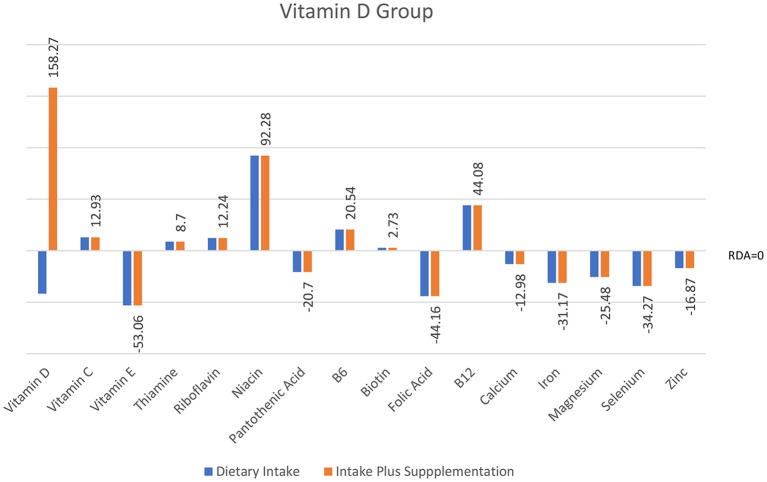
Intake of micronutrients in the Vitamin D group pre- and post-intervention (*n* = 20).

**Figure 4:**
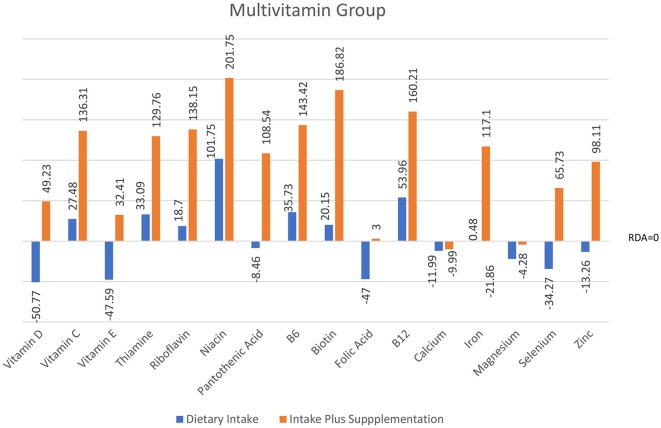
Intake of micronutrients in the Multivitamin group pre- and post-intervention (*n* = 20).

**Figure 5:**
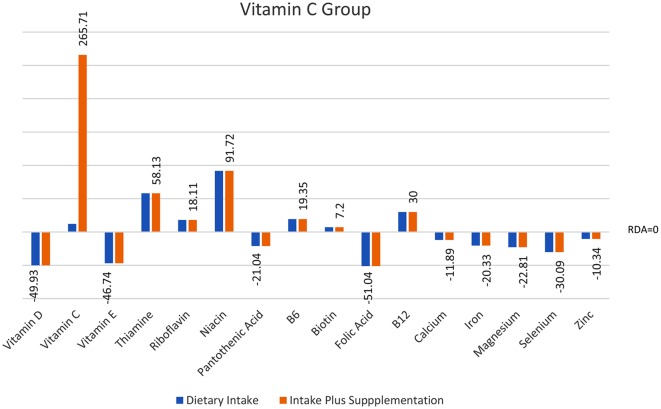
Intake of micronutrients in the Vitamin C group pre- and post-intervention (*n* = 20).

Individual *t*-tests with corrected *p*-value (≤0.01, two-tailed) were conducted to compare cognitive performance from T1 to T2 for each group (see Table 2). These analyses showed improvement in full-scale IQ on the WASI-II (FSIQ-4) in both Multivitamin and Vitamin D groups but not in the Vitamin C group. Immediate and delayed verbal memory and delayed visual memory on the WMS-IV were all significantly improved across the three groups from baseline. In addition, overall performance on the Doors and People, a measure of visual and verbal memory and learning, was significantly improved for all groups. Improvement in working memory (Digit Span) was only seen in the Multivitamin group (see Figures 3–5).

**Table 2:** Paired *t* -tests post intervention by supplementation group.

	Multivitamin			Vitamin D			Vitamin C		
Measure/Function	Time 1 mean (SD)	Time 2 mean (SD)	*t*_(19)_	Time 1 mean (SD)	Time 2 mean (SD)	*t*_(19)_	Time 1 mean (SD)	Time 2 mean (SD)	*t*_(19)_
IQ:									
WASI-II FSIQ-4	110.40 (12.07)	114.05 (10.42)	2.64	113.15 (10.41)	116.50 (11.11)	3.96
Memory:									
WMS Verbal Mem. Immediate	10.80 (2.59)	12.50 (2.59)	2.76	11.60 (2.28)	12.95 (2.56)	3.50	11.75 (2.53)	13.65 (1.84)	3.91
WMS Verbal Mem. Delayed	11.00 (2.63)	13.30 (2.43)	3.63	11.35 (3.13)	13.20 (3.19)	5.07	11.45 (2.61)	14.20 (2.26)	6.82
WMS Visual Repro. Delayed	10.95 (2.35)	14.30 (2.43)	5.95	11.55 (3.65)	14.35 (2.98)	5.43	13.05 (3.25)	15.45 (2.81)	3.29
WMS Symbol Span	11.80 (3.58)	13.65 (3.13)	3.00
Doors and People Overall	12.05 (2.63)	13.70 (2.56)	3.73	13.35 (3.27)	13.80 (2.57)	3.07	12.65 (2.34)	14.45 (2.16)	3.89
Executive Function:									
DKEFS Trail Making Number/Letter Switch (visuo-motor switching)							12.40 (1.31)	12.95 (1.23)	2.77
DKEFS Design Fluency Total (Visual strategy generation)	7.90 (1.55)	9.45 (2.31)	3.81				8.35 (1.39)	9.35 (1.57)	3.16
DKEFS Tower Mean 1st MoveTime (motor planning)	10.95 (2.37)	11.95 (1.50)	3.01
WAIS-III Symbol Search Correct(visuo-motor processing speed)	12.05 (2.67)	13.40 (2.37)	3.18	12.75 (3.52)	13.95 (3.14)	3.27	13.60 (2.04)	14.95 (2.32)	2.97
SRT Explicit Learning	11.15 (5.33)	15.85 (5.27)	3.73						
SRT Implicit Learning	102.74 (73.65)	64.74 (63.25)	3.63						
PANAS Positive Affect									
PANAS Negative Affect									

*All p values ≤ 0.01 (two-tailed)*.

Results of analyses of executive function performance found that the vitamin C group alone showed improvement in visuo-motor switching (Trail Making Switching). Both the Multivitamin and Vitamin C groups showed significant improvements in visual strategy generation (Design Fluency), however, improvements in motor planning (Tower Mean 1st Move Time) were only in the Multivitamin group. All groups showed significant improvements in visuomotor processing speed (Symbol Search) ability over the time course of the study. For the Serial Reaction Time test, only the multivitamin group showed significantly improved explicit awareness of the presented pattern and a significantly reduced reaction time on the implicit learning task.

## Discussion

The present preliminary study had some unexpected findings in that participants were deficient in many micronutrients at baseline, particularly minerals and fat-soluble vitamins. This is a concerning finding for healthy participants in a western first world nation and supports calls for better nutritional awareness regarding diet in the general population (Brown et al., 2011; Beattie et al., 2014; Alkerwi et al., 2015). Micronutrient supplementation completely changed the nutritional profile of the multivitamin group, whilst the two other groups showed more selective nutritional improvements as might be expected. Despite only selective nutritional changes Vitamin C and D groups improved on selective functions over the intervention period. It is possible that these findings indicate the specific beneficial role of selective nutrients, although more data are needed before this can be reliably established.

All groups had better performance on some tasks, notably on measures of verbal and visual memory and visuomotor processing speed, Multivitamin and Vitamin D groups also showing improvements on overall IQ. The Multivitamin group showed significant improvements on tasks of visual strategy generation (along with the Vitamin C group), motor planning, explicit and implicit learning, and working memory. There was no measure where the Vitamin D group alone showed significant improvements.

The finding of improved cognition in the Vitamin C group was unexpected as a review of previous literature in the area (e.g., Arlt et al., 2012) indicated that vitamin C at the dosage administered to participants would not affect cognition, although it is not yet reliably known how small doses of micronutrients might impact brain and cognitive function. There is some research, however, suggesting that increased fruit and vegetable intake is related to improved cognition in the elderly (Gale et al., 1996; Morris et al., 2006). It is, therefore, plausible that in participants with low dietary intake of vegetables and fruit (from food diary entries) increasing levels of vitamin C intake may improve cognition due to the role of ascorbate in underlying neural cellular processes (Harrison and May, 2009). It may be of value to further investigate the role of adequate vitamin C status in cognition across age-ranges.

Following the (relatively short), multivitamin/mineral supplementation period, the Multivitamin group improvements in a number of cognitive functions were observed including working memory, planning and processing speed. Poor micronutrient intake has been demonstrated to negatively affect cognition in childhood and adolescence, and in older populations (Ames, 2006; Nyaradi et al., 2013; Spencer et al., 2017). The evidence from the current study suggests that sub-optimal micronutrient intake may have a negative effect on cognition across the lifespan, rather than being limited to the specific periods previously researched. As there is also evidence that micronutrient insufficiency is a contributing factor in neurodegenerative conditions (Oudshoorn et al., 2008; Balion et al., 2012; Polidori and Schulz, 2014), improvements in cognitive performance seen in those taking supplements could potentially reflect a move towards optimal levels of function, with pre-supplementation performance an indicator of “dulled” performance. If pre-supplementation performance reflects a “dulling” of function in individuals with poor micronutrient intake then this may be an early indicator of the potential for more detrimental cognitive decline later in life, particularly as memory and higher-level cognitive functions are those particularly affected in neurodegenerative diseases of aging (Stopford et al., 2012). In addition, the cognitive functions that showed improvement over the supplementation period are those often affected following traumatic and acquired brain injury and it may be valuable to investigate whether supplementation has similar effects in a clinical sample.

Limitations of this study include the lack of physiological measures of micronutrient status both pre- and post-intervention. Knowledge of micronutrient status, therefore, depended upon self-report of food intake by participants. Although this is a method that is commonly used to assess dietary intake (e.g., Wild et al., 2010; Mak et al., 2012; Ortega et al., 2015), only an estimate of micronutrient intake can be ascertained. In future research, it would be beneficial to gain accurate physiological measures of micronutrient status in addition to food diary data, include a proper control group, and increase the cohort sample size. In this respect, caution must be taken when interpreting these early results. Nevertheless, findings are surprising suggesting that even brief periods of supplementation can enhance cognitive profile, which might offer a new candidate approach to attenuate aging effects on cognition and development of systemic conditions.

## Ethics Statement

This study was carried out in accordance with the recommendations of the Sheffield Hallam University research ethics committee with written informed consent from all participants. All participants gave written informed consent in accordance with the Declaration of Helsinki.

## Author Contributions

RD, LB and CD contributed to the conception and design of the study. RD collected data and performed statistical analyses. RD and LB wrote the first draft of the manuscript. All authors contributed to manuscript revision, read and approved the submitted version.

## Conflict of Interest Statement

The authors declare that the research was conducted in the absence of any commercial or financial relationships that could be construed as a potential conflict of interest.
