# Supplementary material for: Improvement in Cognition Following Double-Blind Randomized Micronutrient Interventions in the General Population
Source: Front Behav Neurosci. 2019 May 28;13:115. doi: 10.3389/fnbeh.2019.00115 (PMC6547837; doi:10.3389/fnbeh.2019.00115)
Supplement: Supplementary file 1 [file Data_Sheet_1.docx]

**Appendix 1 – Composition of the Micronutrient Supplement**

The Boots multivitamin/mineral contained: vitamin A (400µg RE), vitamin D (5µg), vitamin E (12mg α-TE), vitamin C (80mg), thiamine (1.1mg), riboflavin (1.4mg), niacin (16mg NE), vitamin B6 (1.4mg), folic acid (200µg), vitamin B12 (2.5µg), biotin (50µg), pantothenic acid (6mg), vitamin K (75µg), calcium (200mg), iron (14mg), magnesium (60mg), zinc (10mg), iodine (150µg), chromium (40µg), copper (0.5mg), manganese (0.5mg), molybdenum (50µg), selenium (55µg).

The composition of the multi-micronutrient supplement administered included 100% of the Recommended Daily Amount of all micronutrients except for vitamin A and copper (50% RDA), calcium and manganese (23% RDA) and magnesium (16%).
